# Outdoor Physical Activity During the First Wave of the COVID-19 Pandemic. A Comparative Analysis of Government Restrictions in Italy, France, and Germany

**DOI:** 10.3389/fpubh.2021.615745

**Published:** 2021-06-04

**Authors:** Enrico Michelini, Nico Bortoletto, Alessandro Porrovecchio

**Affiliations:** ^1^Department of Sport and Sport Science, Technical University of Dortmund, Dortmund, Germany; ^2^Department of Communication Sciences, University of Teramo, Teramo, Italy; ^3^Univ. Littoral Côte d'Opale, Univ. Lille, Univ. Artois–ULR 7369–URePSSS–Unité de Recherche Pluridisciplinaire Sport Santé Société, Dunkirk, France

**Keywords:** physical activity, COVID-19, sport, coronavirus pandemic, sociology, politics

## Abstract

**Introduction:** Mandated restrictions on outdoor physical activity (PA) during the coronavirus pandemic disrupted the lifeworld of millions of people and led to a contradictory situation. On the one hand, PA was perceived as risky behaviour, as it might facilitate transmission of the virus. On the other hand, while taking precautions, regular PA was an important tool to promote the population's health during the lockdown.

**Methods:** This paper examines the differences in government restrictions on PA in France, Germany, and Italy during the first wave of the COVID-19 pandemic. We draw on techniques of qualitative content analysis and apply a critical theoretical framework to assess the countries' restrictions on PA.

**Results:** Our analysis shows that the restrictions on PA varied in the three countries, in all three countries. This variance is attributed both to differences in the timing and severity of the pandemic in the countries analysed, as well as to the divergence in the relationships between the countries' sport and health systems.

**Conclusion:** At the national level, the variance in restrictions on PA reflect the differences in the spread of the coronavirus and in the health systems' understanding of and approach to PA. The global scientific discourse on the pandemic represents a further key influencing factor. The management of the coronavirus pandemic has demonstrated that the extreme complexity of societies in terms of public health, politics, and the economy pose challenges and unsolvable contradictions.

## Highlights

- Extraordinary interventions on population's lifeworld during the coronavirus pandemic.
- Different national approaches to PA restrictions.
- Emergence of distorted communications and forms of normative decisionism.

## Introduction

On 13 April 2020, a couple of policemen chased, stopped, and fined a man who was jogging alone on an Italian beach with his dog. Numerous media outlets covered this “news” and the footage of this incident, which was filmed from a police helicopter, became an iconic clip. While this fact is *per se* irrelevant, it hyperbolically exemplifies the limitations the coronavirus pandemic imposed on physical activity (PA). This report analyses governments' decisions on PA during the first wave of the COVID-19 pandemic (February to April 2020) in France, Germany, and Italy. Guided by a Habermasian theoretical approach, the critical analysis of these communications lays at the core of this paper. According to recently published research agendas in the sociology of sport (1, 2), the pandemic was widely covered and discussed in both traditional and social media formats. From early on, this content also focused on the implications for sport, exercise, and PA. However, little is known about the present and future impact of the pandemic in this regard.

Over the last 20 years, the accumulation of scientific evidence confirms the benefits of leading an active physical life to maintain and protect one's overall health and well-being at all ages (3). According to the WHO (4), today, physical inactivity and a sedentary lifestyle are the fourth leading cause of death worldwide (5) and continue to pose a major public health challenge. Moreover, sedentary lifestyles lead to physiological disorders, which in turn generate significant health care costs (6). To prevent the spread of such diseases and to improve populations' health, PA promotion has been a key objective of global health strategies and policies for decades (7).

The coronavirus pandemic has radically changed the significance of PA for health, disrupting the PA routines of millions of people worldwide. The mandated restrictions imposed during the first wave of the pandemic significantly impacted PA related to work, commuting, sport and exercise, and has led to a contradictory situation. On the one hand, while not all forms of PA are equally risky, most types were at some point perceived as potentially aiding the spread of COVID-19. On the other hand, while taking precautions, PA remained an important tool to promote the population's health during the lockdown (8–10). Previous pandemic crises caused serious public health consequences that were not only linked to the viral infection *per se*. The indirect consequences on community health have rarely been assessed, however. Studies on the severe acute respiratory syndrome (SARS) epidemic, for example, find that the community in Hong Kong responded by adopting healthier behaviours (11). Some authors argue, however, that the coronavirus pandemic has the potential of further intensifying physical inactivity and sedentary behaviour, which are entrenched in modern western society (12–14).

Despite the scientific consensus on the benefits of PA and the implementation of incentives to promote PA and the engagement of people in more active lifestyles, sedentary behaviour, and physical inactivity were on the rise before the outbreak of the pandemic, especially in high-income countries (15). Many scholars, who have analysed public health data during the pandemic, stress that policymakers should not ignore modifiable lifestyle factors, such as dieting and PA (16), and mental health issues (17).

Against this background, the aim of this report is to analyse the differences in government restrictions on PA in France, Germany, and Italy during the first wave of the COVID-19 pandemic. The following sections describe our theoretical framework and methodological approach. We then present our results and conclude the paper with a discussion of our findings.

## Materials and Methods

Our view of the world *(Weltanschauung*) and theoretical approach is based on critical theory. Habermas (18) distinguishes between lifeworld and system. The former refers to the domain of shared understandings and a social horizon of everyday events, while the latter covers the domain of scientific and technical interests, guided by rational logic. PA's practise is a classic lifeworld domain that emerges from people's daily routines, sociocultural context and individual preferences. Controlling PA is one of those cases in which systemic logic penetrates—or rather colonises (19)—the symbolic reproduction of the lifeworld. Despite being rational by definition, systemic logics are manifold and may be divergent, since they pursue different goals.

The coronavirus pandemic has exacerbated the *steering problem*, making the system untenable because of internal contradictions that manifest themselves in the breakdown of normative structures (20). Society evolved through a set of communicative actions that encompasses and structures the lifeworld of actors (21). According to the *Theory of Communicative Action* (18), any act of communication must encompass four “validity claims”: comprehensibility, sincerity, legitimacy, and truth. An ideal speech situation satisfies all requirements for mutual understanding. The communication of a political institution, in particular, should not violate the validity claims, should not manipulate or be systematically distorted. These crucial checkpoint criteria have been further developed in Habermas' work *Between Fact and Norms* (22). He argues that norms are only valid if the recipient population accepts them and when this acceptance is based on the above-mentioned rational discourse (21). Ideally, as many people as possible must be informed and involved in the public debate.

We used this theoretical framework to interpret a catalogue of selected government communications on PA in Italy, France, and Germany during the first wave of the COVID-19 pandemic. The early months of 2020 were amongst the most dramatic for Europe due to the novelty of the coronavirus, its severity and the high infection rate. As the leading authority in our case countries, government-issued documents were analysed for our study. In contrast with a previously published review of international public health responses to the COVID-19 outbreak (23–25), our analytical strategy focuses on a small sample and applies the most similar stems design (26). Focusing on three conservative welfare states (27), which are highly populated, economically relevant, and geographically close, allows us to conduct an in-depth analysis and comparison.

The documents listed in Table 1 are the primary source used for our sociological analysis. The review of these documents was loosely oriented around a qualitative content analysis, a systematic and flexible empirical method to examine the meaning behind data (28). Therefore, the content of the documents was selected, reduced and successively assigned to the categories of a coding system. Nevertheless, the interpretation of the material through the above-explained critical theoretical framework lies at the core of this study. However, we adapted part of the systematic approach to identify, analyse, compare, and criticise the narratives that lie at the core of our three case studies. This approach was followed to provide reliable insights to the question: “How did governments regulate PA practise during the coronavirus pandemic?”

**Table 1:** List of documents analysed.

**Nation**	**Headline of the document**	**Issuing date**
Italy^a^	Decision of the President of the Council of Ministers	26/04/2020
	Decision of the President of the Council of Ministers	10/04/2020
	Decision of the President of the Council of Ministers	01/04/2020
	Ministry of Health Ordinance	20/3/2020
	Decision of the President of the Council of Ministers	08/03/2020
	Decision of the President of the Council of Ministers	04/03/2020
	Decision of the President of the Council of Ministers	01/03/2020
	Decision of the President of the Council of Ministers	23/02/2020
	Ministry of Health Ordinance	23/02/2020
France	Prime Minister's speech introducing the deconfinement plan	28/04/2020
	Decree No. 220-423 supplementing Decree No. 220-293 prescribing the general measures against the COVID-19 epidemic	14/04/2020
	Decree No. 2020-293 prescribing the general measures against the COVID-19 epidemic	23/03/2020
	Emergency Law No. 2020-290 to mitigate the COVID-19 epidemic	23/03/2020
	Decree No. 2020-260 regulating travel in the context of the fight against the spread of the COVID-19 virus	16/03/2020
	Order for various measures to control the spread of the COVID-19 virus	14/03/2020
Germany	Telephonic conference of the Federal Chancellor and the heads of the federal states	15/04/2020
	Discussion between the Chancellor and the heads of the federal states	22/03/2020
	Agreement between the government and the heads of the federal states	16/03/2020
	Guidelines to slow the spread of the coronavirus	16/03/2020
	Discussion between the Chancellor and the heads of the federal states	12/03/2020

a *In Italy the total number of dispositions, decisions and laws relating to COVID-19 up to 30 April 2020 amounted to 235, coming from various sources, including Parliament, the Council of Ministers' Presidency, Ministries, Agencies and ad hoc Commissioners. https://www.gazzettaufficiale.it/dettaglioArea/12 [only in Italian]*.

## Results

The following sections briefly summarise—in chronological order and in a comparative perspective—the political decisions implemented in Italy, France, and Germany to regulate PA practise. The discussion critically evaluates the results.

### Italy

All of the Italian Ministry of Health's National Health Plans have included the promotion of PA as one of the key public health goals. Additionally, over 14 million Italians of all ages claim that they regularly engage in sports (29). This suggests that the implementation of a strict lockdown, which in Europe was dubbed the “Italian lockdown,” raised several issues.

The coronavirus began to spread across Italy at the end of January 2020. The highly populated region of Lombardy was hit particularly hard right from the beginning. On 31 January, the central government declared a state of emergency, mainly for economic reasons, pursuant to the law on civil contingencies (Civil Protection Code: CPC), which does not require parliamentary approbation. Such a declaration allows ministers to adopt exceptional measures in case of natural disasters, such as earthquakes and floods, but it is questionable whether limitations on civil liberties are justified for dealing with a pandemic. Despite this emergency declaration, no significant measures were introduced in the following weeks. On 23 February 2020, some municipalities in Lombardy were locked down and the Ministry of Health suspended all sport events in most parts of northern Italy.

On 1 March 2020, a decision of the Prime Minister confirmed the previous measures, which had ordered the closing of gyms, swimming pools, and other sport facilities in Lombardy and parts of the Emilia Region. Skiing was still permitted under the condition of “social distancing,” and 3 days later, it was decided that also “sport for all” activities (outdoor and indoor) were allowed only under this condition. On 8 March, Lombardy, Emilia, parts of the Veneto and the Piedmont regions were locked down, and all sport competitions were prohibited. The skiing areas in those regions were also closed, causing a tourist migration to the adjacent ski areas. On 20 March, the Ministry of Health ordered the closure of parks and green spaces, banning outdoor play and recreation. PA (but not jogging) was allowed within a 200-metre radius from home, always respecting the social distancing rules. On 26 April 2020, the Prime Minister announced that the parks and green spaces would reopen as of 4 May, and that sport for all—under the condition of social distancing, of course—would be permitted, and that professional athletes in some sport disciplines could resume training under given guidelines developed by the medical commission of the Italian Olympic Committee.

During the lockdown, Italians generally refrained from PA, limiting PA to either exercising at home or taking very short walks in close proximity to their homes (30). Some people even dared to engage in sport, at the risk of being reported to the police in a kind of *untorn* modern witch hunt.

### France

To understand the situation in France, it must be framed within the context of recent government policies on health [“health democracy” (31)], and the fight against sedentary or inactive lifestyles: public health issues have been at the heart of the French government's agenda. Among the measures implemented in this regard, the health education programme “National Health and Nutrition Plan” (developed by the Agency for Health Food Safety in 2001) states that “people who regularly engage in PA have a lower risk of developing long-lasting diseases, regardless of their eating patterns and lifestyle habits” (32). Today, the 2019–2024 National Sport Health Strategy aims to reinforce this paradigm to fully recognise physical and sport activities as factors of physical and mental health, and to propose solutions that allow for such activities to be carried out under safe conditions.

The COVID-19 pandemic reached France on 24 January 2020, when the first case in Europe was confirmed in Bordeaux. However, only at noon on 17 March 2020, in the wake of the crisis in Italy, did France enter a confinement mechanism, which included the implementation of specific measures. The Ministerial Order of 14 March closed all sport facilities. The Decree of 16 March introduced the concept of self-certification, and the Decree and subsequently the Law of 23 March “normalised” the applicable regulations: people were allowed to take short walks, which were limited to 1 h daily within a maximum radius of one kilometre from home. Individuals could also engage in “basic PA,” respecting the rules on social distancing, but any collective “sport activity” and proximity to others was forbidden. People could only take walks with those who lived in the same household; walking a pet was also allowed. A self-certification form was drawn up that differed from Italy's and included additional “reasons” for leaving one's home: individuals had the option of leaving their home to run or to take a walk, either alone, or with family members.

The Decree of 14 April 2020 extended the provisions of both the Decree and Law of 23 March. Toward the end of that month, on 28 April, the French Prime Minister gave a formal address about the country's re-opening strategy, explaining that it would be gradual, and that it would vary depending on the region. In the most affected ones (the “red zones”), parks would remain closed, but individual PA (excluding sport and collective PA) would be possible, even beyond the initially established one kilometre radius from home.

### Germany

As is the case in many developed countries, the promotion of PA is an important objective of German health policies. Amongst other initiatives, the National Action Plan IN FORM (33), launched in 2008, explicitly draws on international health promotion guidelines and aims to provide “support for changes in behaviour through information and motivation, and the further development of health-promoting structures.

The first COVID-19 case was identified near Munich, Bavaria, on 27 January 2020. The Infection Protection Act (34) establishes that the state may restrict or temporarily suspend the basic rights of the population. During the coronavirus pandemic, fundamental rights such as personal freedom, freedom of assembly, and the right to bodily integrity were restricted. However, the restrictions mandated in Germany were moderately permissive compared to those imposed in other countries (for example, in Italy). Following a preparatory political meeting, the German government adopted official guidelines to contain the spread of COVID-19 on 16 March 2020. These guidelines were supplemented and replaced by new and more restrictive measures on 22 March. Individual outdoor sport and PA were permitted throughout the lockdown period. The system of self-certification was not used in Germany. The initial measures imposed the closure of all sport facilities (public and private, outdoor, and indoor). On the same day, the federal government and the heads of the federal states agreed to an exceptionally uniform response to the coronavirus pandemic in Germany. The federal government and federal states jointly decided on 15 April to extend the applicable restrictions until 3 May, but at the same time, allowed small businesses to re-open and gradually re-opened educational facilities and religious buildings. The loosening of these general restrictions was applied with minor deviations in the German states. In Rhineland-Palatinate, for example, some sport facilities were re-opened. This, and specifically the question about the requirement to wear protective masks, reignited the debate on the need for decision-making at the central level.

## Discussion

The coronavirus pandemic dramatically intensified government intervention in the lifeworld of populations (2). Heeding the advice of the medical-scientific domain, national, regional, and local political levels implemented decisions, on occasion also using coercive methods. Different levels of PA restrictions identified in the three countries during the first wave of the pandemic reflect both the differences in timing and severity of the pandemic in the countries analysed, but the restrictions hinged on the same rational logic underlying the global epidemiological discourse (Table 2).

**Table 2:** Summary of results^a^.

**Country**	**First reported infection**	**First PA restriction**	**Loosening of PA restrictions**	**Intensity of PA restriction**	**Coupling of health-sport systems**	**Mortality excess (first wave)**
Italy	31-01-2020	23-2-2020	18-05-2020	High	Moderate	High
France	24-01-2020	14-03-2020	28-04-2020	Moderate	Strict	Moderate
Germany	27-01-2020	16-03-2020	15-04-2020	Moderate	Strict	Low

a *The “intensity”, “coupling” and “mortality” indicators have no analytical value. They only provide a synopsis of the report. The “mortality excess wave” indicator measures the deviation in mortality from the expected level. The table does not consider absolute values and compares instead the data reported among the three countries. Information based on Eurostat data: https://ec.europa.eu/eurostat/databrowser/view/DEMO_MEXRT__custom_342641/bookmark/table?lang=en&bookmarkId=7c411664-aa81-460c-aa40-22472512fe8b*.

Divergent relationships between the sport and health systems may also be an explanation for the differences. In France and Germany, for example, PA is treated as a key component of the health system and is perceived as a protective factor. In Italy, PA was ultimately not considered crucial and, paradoxically, represented an element of potential corrosion of social consensus on the lockdown.

From a Habermasian point of view, the pandemic poses major challenges (35) in terms of carrying out swift and relevant interventions in the context of complex and differentiated societies. Due to the novelty of the situation and structural problems of democratic decision-making procedures, the differences were also attributable to partly divergent goals in public health, politics, and the economy. In the best-case scenario, broad consensus on the regulation of liberties and freedom would have been reached based on rational discourse. Instead, the public sphere, in the sense of an open, non-coerced debate, seems to have followed the path illustrated by Habermas in the sixties: a progressive trivialisation that seems to severely hamper the formation of public opinion, leading again to the so called “refeudalisation” of reality, impeding the building of consensus and, consequently, leading to forms of normative decisionism that deviate from ideal communications, are not fully transparent and only partially admissible in the reality of Western democracies (36). In the case of Italy, for instance, the huge number of “Decisions of the President of the Ministers Council”—normally a rarely used normative instrument—was employed as a means of restricting personal freedoms, with very limited involvement of Parliament, leading to intense criticism, also from public law scholars.^1^

The problem, in particular, of truthfulness emerges in relation to the rapid implementation of political decisions based on scientific knowledge. In an ideal speech situation, all arguments presented in the discussion should be factually correct, verifiable, and scientifically based. Yet several issues affected the truth of scientific rationality beyond the justifications of the approach to contain the pandemic. From an epistemological perspective (37), scientific knowledge on COVID-19 is still partly conjectural or hypothetical since the virus and its transmission are new to the scientific community. Consequently, scientific assumptions were decidedly followed, even though they were far from being conclusively proven and could be and were falsified in the short-term future. Moreover, determining the indirect impacts of the measures adopted was excessively complex and may only emerge in the mid to long term. This certainly applies to the restrictions on PA, whose risk assumptions did not rely on solid scientific evidence.

Aside from these inherent limitations, scientific knowledge is sometimes used misleadingly to implement political decisions. This was particularly evident in the context of the strict “Italian lockdown.” Despite the low risk for spreading the virus, individual outdoor running was completely prohibited in Italy. The rationale for this was the concern that too many people would have used outdoor running as a way to evade the lockdown restrictions. In other words, scientific evidence was misrepresented to support strategic aims. The prohibition of outdoor PA is an extraordinary interference of the system on the population's lifeworld. Because of the state of emergency, and with the legitimation of the medical-scientific domain, the political system was successful in exerting its power. Indeed, leveraging on medical, moral and patriotic argumentations, the restrictions during the first wave of the COVID-19 pandemic were met with less resistance by the population in the three countries covered in our study. Protests against the restrictions arose during the second wave and were associated with the prevalence of “pandemic fatigue” in the general population rather than with the severity of the restrictions (38).

Despite these critical considerations of the trade-offs between the benefits and detriments associated with the restrictions on PA, the dramatic and rapidly evolving course of the pandemic in the period considered here was acknowledged as being perhaps the most serious challenge Europe has faced since the Second World War. In this context, policymakers may have not taken adequate heed of the risks associated with the lockdown, but they did not take the decision lightly, and had to juggle the different risks. In hyper-complex societies and in critical situations, political communication cannot be ideal. Nonetheless, some recommendations can be formulated. Specifically, public communication needs to be separated from regulatory activity. The former must be disseminated unambiguously, promptly and adequately with regard to the situation at hand. The rapid dissemination of information through different media (e.g., social media, television, or government websites) may have a negative impact on the information's reception. Furthermore, regulatory activity must take account of the temporal dimension of the emergency through the possible minimal use of extraordinary regulatory instruments. The habitual (democratic and/or federal) decision processes through the entrusted political bodies should be restored as soon as the emergency permits.

To conclude, the coronavirus pandemic is an ongoing critical event, which indubitably needs to be further analysed and reflected upon based on different perspectives. Critical sociological research on health and PA policies may contribute to reflections on and to safeguarding the rationality of the political discourse (21). While pursuing this aim, this report constitutes only a first and explorative step in this direction. Amongst other limitations, the available data do not permit an in-depth discussion on other important factors which may have also played an influential role in the governments' choices. This, as well as other aspects related to the question “How will sport, exercise and PA change in the aftermath of the pandemic?” represent interesting phenomena for sociological analysis of PA, and we encourage inquiries into these issues in the future.

## Data Availability Statement

The original contributions presented in the study are included in the article/supplementary material, further inquiries can be directed to the corresponding author/s.

## Author Contributions

All authors listed have made a substantial, direct and intellectual contribution to the work, and approved it for publication.

## Conflict of Interest

The authors declare that the research was conducted in the absence of any commercial or financial relationships that could be construed as a potential conflict of interest.

## Footnotes

^1^Among the most relevant articles, see [in Italian]: Azzariti, G. (2020). I limiti costituzionali della situazione d'emergenza provocata dal Covid-19. Questione Giustizia, 27; Massa Pinto, I. (2020). La tremendissima lezione del Covid-19 (anche) ai giuristi. Fiat iustitia et pereat mundus oppure Fiat iustitia ne pereat mundus?, Questione giustizia, 18; Civinini, M. G., & Scarselli, G. (2020). Emergenza sanitaria. Dubbi di costituzionalità di un giudice e di un avvocato. Questione Giustizia, 14. [in English]: Simoni A. (2020), Populist legal strategies and enforcement discretion in Italy in the COVID-19 emergency, in Rister de Sousa Lima et al. (eds.), Covid-19 e os impactos no direito: Mercado, Estado, Trabalho, Família, Contratos e Cidadania, Almedina, Brazil.
